# Application of SGLT-2 inhibitors in non-diabetic CKD: mechanisms, efficacy, and safety

**DOI:** 10.3389/fmed.2025.1574693

**Published:** 2025-07-01

**Authors:** Bing Zhang, Liufei Deng

**Affiliations:** Department of Nephrology, Caidian District People's Hospital, Wuhan, China

**Keywords:** sodium-glucose cotransporter 2 inhibitors, safety assessment, clinical efficacy, non-diabetic chronic kidney disease, renal protection mechanisms

## Abstract

Chronic kidney disease (CKD) represents a major global public health challenge, significantly impacting patients’ quality of life and placing a heavy burden on healthcare systems. While diabetes and hypertension are the primary risk factors for CKD, non-diabetic CKD also constitutes a significant proportion, with its complex pathological mechanisms necessitating the development of novel therapeutic strategies. Sodium-glucose cotransporter 2 (SGLT-2) inhibitors, initially developed for diabetes management, have recently demonstrated remarkable renal and cardiovascular protective effects in patients with non-diabetic CKD. SGLT-2 inhibitors exert their effects through multiple mechanisms, including reactivating the tubulo-glomerular feedback, reducing glomerular pressure and filtration rate, decreasing proteinuria, inhibiting inflammation and fibrosis, and improving systemic metabolic parameters such as lowering blood pressure, uric acid levels, and body weight. These effects not only slow the progression of kidney function decline but also significantly reduce the risk of end-stage renal disease (ESRD) and cardiovascular events. Landmark clinical trials such as DAPA-CKD, CREDENCE, and EMPA-KIDNEY provide strong scientific evidence supporting the use of SGLT-2 inhibitors in non-diabetic CKD, demonstrating their broad clinical benefits and excellent safety profile. Despite potential adverse effects such as urinary tract infections, hypotension, and diabetic ketoacidosis, appropriate patient selection and personalized treatment strategies can effectively manage these risks. The multi-system effects of SGLT-2 inhibitors not only expand their clinical indications but also offer new hope for the comprehensive management of non-diabetic CKD patients, with significant clinical implications and broad future application potential.

## 1 Introduction

Chronic kidney disease (CKD) is a global public health issue that poses a significant threat to healthcare systems and patient quality of life due to its high incidence and disability rates. According to the World Health Organization (WHO), approximately 8–16% of adults worldwide are affected by CKD, with a particularly high prevalence in high-income countries (1). CKD not only increases the risk of end-stage renal disease (ESRD) but also significantly raises the incidence of cardiovascular disease, diabetes, and other metabolic disorders, leading to a substantial decline in overall health and quality of life (2).

The causes of CKD are diverse, with diabetes and hypertension being the two primary risk factors. However, non-diabetic CKD (Non-Diabetic CKD) also plays a significant role in clinical practice. Its pathological mechanisms are complex, involving a combination of genetic, environmental, and metabolic factors (3). Patients with non-diabetic CKD often experience pathological changes such as inflammation, oxidative stress, fibrosis, and hyperfiltration, which lead to progressive kidney dysfunction. Currently, treatment options for non-diabetic CKD are limited, and there is an urgent need to identify new, effective therapies and strategies to slow disease progression and improve patient outcomes (4).

In recent years, sodium-glucose cotransporter 2 (SGLT-2) inhibitors have gained attention as a novel class of antidiabetic drugs, particularly due to their significant efficacy in diabetic nephropathy (5). Initially, SGLT-2 inhibitors work by inhibiting glucose reabsorption in the proximal renal tubule, lowering blood sugar levels and improving metabolic conditions in diabetic patients (6). However, with the accumulation of clinical studies, SGLT-2 inhibitors have shown remarkable renal and cardiovascular protective effects in non-diabetic CKD patients, significantly expanding their clinical applications and value (7). This discovery not only provides new treatment options for non-diabetic CKD patients but also offers renewed hope for the comprehensive management of CKD.

SGLT-2 inhibitors exert their protective effects through multiple mechanisms, including reducing glomerular pressure, decreasing proteinuria, inhibiting inflammation and fibrosis, and improving systemic metabolic conditions (8). These pharmacological actions not only effectively delay the deterioration of kidney function but also significantly reduce the risk of ESRD and cardiovascular events. Landmark clinical trials such as DAPA-CKD, CREDENCE, and EMPA-KIDNEY provide solid scientific evidence supporting the use of SGLT-2 inhibitors in non-diabetic CKD patients, confirming their broad clinical benefits and excellent safety profile (9, 10).

Although SGLT-2 inhibitors show great potential in treating non-diabetic CKD, their use requires careful management of potential side effects, such as urinary tract infections, hypotension, and diabetic ketoacidosis (11). Furthermore, responses to SGLT-2 inhibitors may vary across different kidney disease subtypes, and further research and clinical trials are needed to explore the optimal application strategies in specific populations and disease contexts (12).

This review aims to systematically summarize the mechanisms of action, clinical efficacy, safety assessment, and potential applications of SGLT-2 inhibitors in non-diabetic CKD and other kidney disease subtypes. By thoroughly analyzing existing research, this review provides scientific evidence for clinical practice, aiming to optimize treatment protocols and improve the prognosis and quality of life for non-diabetic CKD patients (13). Additionally, it explores future research directions and challenges, with the goal of advancing the widespread application and further development of SGLT-2 inhibitors in the management of CKD and related disorders.

## 2 Mechanism of action of SGLT-2 inhibitors

In recent years, SGLT-2 inhibitors have demonstrated significant renal protective effects in the treatment of CKD. By reducing glomerular pressure and proteinuria, they effectively alleviate mechanical stress on the kidneys and slow the progression of renal dysfunction (14). Additionally, these drugs exert dual anti-inflammatory and anti-fibrotic effects by modulating key signaling pathways, further inhibiting the progression of CKD. At the same time, SGLT-2 inhibitors positively impact overall health by improving systemic metabolic conditions, including lowering blood pressure, uric acid levels, and body weight (15). The synergistic effects of these multiple mechanisms not only enhance patients’ quality of life but also provide new therapeutic strategies for the comprehensive management of non-diabetic CKD, highlighting the important role and broad application prospects of SGLT-2 inhibitors in modern nephrology (16).

### 2.1 Direct renal protective effects

#### 2.1.1 Reduction of glomerular pressure

Glomerular hyperfiltration is a significant pathological feature of CKD progression, particularly prominent in the early stages of the disease. The development of hyperfiltration is closely associated with excessive reabsorption of sodium (Na⁺) and glucose in the proximal renal tubules (17). This excessive reabsorption reduces the sodium load reaching the distal tubules, impairing the ability of the macula densa to detect sodium concentration, thereby inhibiting normal tubuloglomerular feedback and causing dilation of the afferent arteriole (18). Dilation of the afferent arteriole increases glomerular pressure, and prolonged hyperfiltration exerts excessive mechanical stress on the glomerular filtration barrier, ultimately leading to podocyte injury, basement membrane expansion, and glomerulosclerosis (19). SGLT-2 inhibitors block the reabsorption of sodium and glucose in the proximal tubules, significantly increasing the sodium load reaching the distal tubules, thereby reactivating the tubuloglomerular feedback mechanism. When the macula densa detects an increase in sodium concentration, it releases signaling molecules such as adenosine, which causes constriction of the afferent arteriole, thereby reducing blood flow into the glomerulus (20). Ultimately, this mechanism reduces glomerular pressure and filtration rate, significantly alleviating the mechanical damage caused by hyperfiltration to the glomerulus. As glomerular pressure decreases, stress on podocytes and the basement membrane is reduced, restoring their barrier function and further preventing proteinuria and the progression of glomerulosclerosis (21). Clinical studies support the significant effects of this mechanism. For example, the DAPA-CKD study showed that patients treated with SGLT-2 inhibitors had a significantly slower decline in glomerular filtration rate (GFR), particularly those in a state of hyperfiltration (22). This effect has been validated in both diabetic and non-diabetic CKD patients, demonstrating the broad applicability of SGLT-2 inhibitors in regulating glomerular pressure.

#### 2.1.2 Reduction of proteinuria

Proteinuria is a key marker of CKD, and its degree not only reflects the severity of renal damage but also accelerates disease progression. Proteinuria is primarily associated with damage to the glomerular filtration barrier (23). The glomerular filtration barrier consists of podocytes, the basement membrane, and endothelial cells. Under hyperfiltration, excessive mechanical stress and inflammatory factors lead to damage to the barrier, making it easier for proteins (such as albumin) to filter from the blood into the urine (24). The proteins in the urine further activate tubular epithelial cells, inducing the secretion of inflammatory factors and pro-fibrotic factors. These molecular signals promote inflammation and fibrosis in the renal interstitium, thereby accelerating the deterioration of kidney function (25).

SGLT-2 inhibitors reduce glomerular pressure, fundamentally decreasing pressure-induced damage to the glomerular filtration barrier, and significantly reducing proteinuria. Furthermore, SGLT-2 inhibitors mitigate inflammation and fibrosis associated with proteinuria through various indirect mechanisms (26). In clinical trials, SGLT-2 inhibitors significantly reduced urine protein levels in patients with diabetic and non-diabetic nephropathy, validating their clinical efficacy in reducing proteinuria (27).

### 2.2 SGLT-2 inhibitors in non-diabetic CKD renal injury protection and mechanisms

The therapeutic effects of SGLT-2 inhibitors in non-diabetic CKD patients extend beyond their glucose-lowering actions. These drugs exert significant renal protective effects by modulating several key signaling pathways, including AMP-activated protein kinase (AMPK), hypoxia-inducible factor 1-alpha (HIF-1α), (transforming growth factor *β*) TGF-β, nuclear factor-kappa B (NF-κB), and the renin-angiotensin-aldosterone system (RAAS) pathways (28).

Firstly, SGLT-2 inhibitors activate the AMPK signaling pathway. AMPK serves as a major energy sensor within cells, and when cellular energy levels drop, AMPK is activated (29). Upon activation, AMPK phosphorylates several downstream target proteins to regulate various metabolic processes (30). Specifically, SGLT-2 inhibitors inhibit glucose reabsorption in the renal tubules, reducing intracellular glucose and ATP production, which raises the AMP/ATP ratio and activates AMPK. Some studies suggest that SGLT-2 inhibitors may also directly interact with the AMPK activation complex to further enhance its activity (31). Activated AMPK promotes fatty acid oxidation, increases the expression of carnitine palmitoyltransferase 1, reduces lipid accumulation, and mitigates oxidative stress related to lipid metabolism. Additionally, AMPK inhibits the mechanistic target of rapamycin (mTOR) signaling pathway, reducing protein synthesis and cell proliferation while promoting autophagy to clear damaged organelles and proteins, alleviating cellular stress and damage. Furthermore, AMPK activation suppresses the TGF-*β* and NF-κB pathways, reducing the production of pro-fibrotic and inflammatory factors, thereby alleviating renal fibrosis and inflammation (32). In addition to its role in metabolic regulation, AMPK activation plays a significant role in immune regulation. AMPK activation enhances the function of Foxo transcription factors, such as Foxo1 and Foxo3, which in turn promotes the proliferation of regulatory T cells. These Treg cells are essential for suppressing chronic inflammation. Furthermore, this process upregulates FOXP3 expression, thereby further reducing chronic inflammation associated with type 2 diabetes and its complications. Therefore, AMPK activation not only improves metabolic function but also offers potential therapeutic benefits in managing inflammation-induced complications (33).

Secondly, SGLT-2 inhibitors regulate the HIF-1α signaling pathway, improving the hypoxic state of renal tubules and stabilizing HIF-1α to function under low-oxygen conditions (34). Under normal oxygen conditions, HIF-1α is rapidly degraded, but in hypoxic conditions, HIF-1α is stabilized and translocated to the nucleus, where it promotes the expression of downstream genes, such as vascular endothelial growth factor (VEGF), to stimulate angiogenesis, improve renal microcirculation, and enhance tissue oxygen supply (35). HIF-1α also regulates the expression of glucose transporter 1 (GLUT1) and glycolytic enzymes, enhancing energy production under hypoxic conditions and improving the survival and function of renal tubular cells. Additionally, HIF-1α promotes the expression of antioxidant enzymes such as superoxide dismutase (SOD), reducing oxidative stress and protecting the integrity of renal tubular cell functions (36). By reducing renal tubular energy consumption and oxygen demand, SGLT-2 inhibitors improve local oxygen supply and stabilize HIF-1α, enhancing cellular resistance to hypoxia and promoting angiogenesis (34).

In the TGF-*β* signaling pathway, TGF-β is a key pro-fibrotic factor in fibrosis processes, which activates the Smad2/3 signaling pathway, promoting the transformation of fibroblasts into myofibroblasts and increasing extracellular matrix (ECM) deposition, leading to tissue fibrosis (37). SGLT-2 inhibitors suppress excessive TGF-*β* expression by reducing proteinuria and oxidative stress. Specifically, by inhibiting the accumulation of filtered proteins in the glomeruli, SGLT-2 inhibitors reduce proteinuria and, thereby, decrease renal oxidative stress, indirectly inhibiting TGF-*β* expression (38). Furthermore, AMPK activation further suppresses Smad2/3 phosphorylation, blocking downstream fibrotic signaling. SGLT-2 inhibitors also upregulate bone morphogenetic protein-7 (BMP-7), which has anti-fibrotic effects, restoring the normal phenotype of renal tubular epithelial cells and reducing ECM deposition, effectively slowing the progression of renal interstitial fibrosis (39).

The NF-κB signaling pathway plays a central role in inflammation, and its activation promotes the expression of pro-inflammatory factors such as tumor necrosis factor *α* (TNF-α) and interleukin 6 (IL-6), mediating chronic inflammatory responses. SGLT-2 inhibitors reduce reactive oxygen species (ROS) production, lower the phosphorylation and degradation of IκB, and inhibit NF-κB nuclear translocation and activation (40). Specifically, by promoting fatty acid oxidation and reducing lipid accumulation, SGLT-2 inhibitors decrease ROS generation, mitigating oxidative damage to cells, thus slowing IκB degradation (41). This preserves the binding of NF-κB to IκB, preventing its translocation to the nucleus. Additionally, by inhibiting NF-κB activity, SGLT-2 inhibitors reduce the expression of pro-inflammatory cytokines such as TNF-*α* and IL-6, lower the expression of inflammation-related adhesion molecules such as ICAM-1 and VCAM-1, and reduce the infiltration of inflammatory cells into the renal tubulointerstitium, alleviating renal inflammation and reducing the risk of fibrosis (42).

Moreover, SGLT-2 inhibitors regulate the RAAS signaling pathway, further protecting renal health. RAAS plays a critical role in regulating blood pressure and fluid balance, and its overactivation is closely associated with hypertension, proteinuria, and renal fibrosis (43). SGLT-2 inhibitors inhibit sodium-glucose co-transport in the proximal tubules, increase sodium excretion, and reduce glomerular afferent flow, alleviating glomerular hyperfiltration. Additionally, through their diuretic effect, SGLT-2 inhibitors decrease blood volume and peripheral vascular resistance, mildly lowering blood pressure and reducing the burden on the heart and kidneys (44). These effects, through a negative feedback mechanism, suppress renin secretion, leading to decreased production of angiotensin II and aldosterone, and inhibiting excessive RAAS activation (45). Specifically, reducing Ang II production lowers its activation of AT1 receptors, alleviating vasoconstriction and sodium reabsorption, thereby lowering blood pressure. Furthermore, reducing pro-fibrotic factor expression protects the glomeruli, reduces proteinuria, and delays the deterioration of renal function (46).

**Figure 1:**
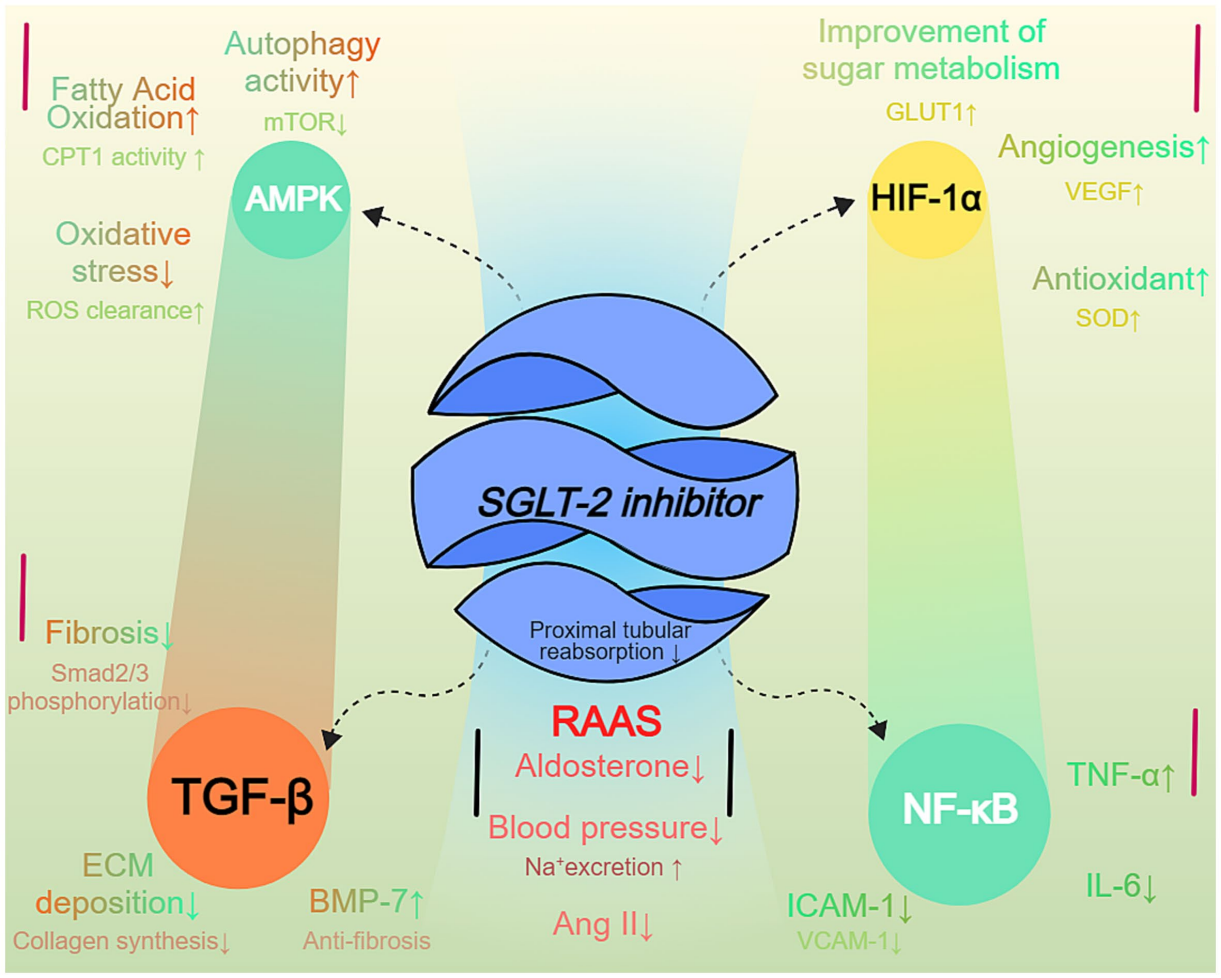
SGLT-2 inhibitor-mediated signaling pathways in non-diabetic CKD. SGLT-2 inhibition modulates multiple signaling pathways in chronic kidney disease (CKD). The diagram shows five key pathways: AMPK activation improves metabolic efficiency and reduces oxidative stress; HIF-1α stabilization enhances glucose metabolism and angiogenesis; TGF-β suppression reduces fibrosis; NF-κB modulation decreases inflammation; and RAAS inhibition lowers blood pressure and improves sodium homeostasis. Arrows indicate pathway activation or inhibition.

Through the synergistic action of multiple signaling pathways, SGLT-2 inhibitors comprehensively improve renal metabolic status, reduce oxidative stress and inflammation, and inhibit fibrosis, thereby effectively delaying the progression of non-diabetic CKD (47). These complex molecular mechanisms not only highlight the multiple effects of SGLT-2 inhibitors in renal protection but also provide new strategies and directions for the treatment of CKD, underscoring their importance in modern nephrology (48). Clinical studies, such as the DAPA-CKD and CREDENCE trials, further validate the efficacy of these mechanisms, demonstrating that SGLT-2 inhibitors significantly reduce the risk of renal function deterioration in non-diabetic CKD patients and improve overall quality of life, making them an important therapeutic option in CKD management (Figure 1).

### 2.3 Anti-inflammatory and anti-fibrotic effects

In CKD, renal inflammation is a critical factor, often associated with the overproduction of pro-inflammatory cytokines such as TNF-*α* and IL-6. These cytokines activate immune cells, triggering inflammation in the kidneys, which further damages the renal tubules and interstitial tissue, ultimately accelerating the decline of kidney function (49).

First, SGLT-2 inhibitors reduce the synthesis of pro-inflammatory cytokines by inhibiting the NF-κB signaling pathway. NF-κB is a key transcription factor involved in numerous inflammatory responses. Studies have shown that SGLT-2 inhibitors reduce NF-κB activity in the kidneys, thereby decreasing the expression of pro-inflammatory cytokines like TNF-*α* and IL-6, and alleviating renal inflammation (50). Additionally, SGLT-2 inhibitors suppress the inflammatory response by reducing the infiltration of M1 macrophages. M1 macrophages are key cells in renal inflammation, secreting large amounts of pro-inflammatory cytokines. SGLT-2 inhibitors reduce the migration of these cells, lowering local inflammation levels (51). Moreover, SGLT-2 inhibitors may further suppress inflammation by enhancing the expression of anti-inflammatory factors, such as IL-10. IL-10, as an anti-inflammatory cytokine, inhibits the activation of inflammatory cells and reduces renal inflammatory damage (10, 52).

Renal fibrosis is a significant feature in the progression of CKD, often caused by tubular injury, persistent inflammation, and the activation of fibroblasts. TGF-*β* is a key factor in the fibrotic process. It activates the downstream Smad signaling pathway, leading to the synthesis of matrix components such as collagen, which initiates fibrosis. Connective tissue growth factor (CTGF) is an effector molecule of the TGF-*β* pathway and further promotes fibroblast proliferation and collagen deposition, accelerating the fibrotic process (53).

The anti-fibrotic effects of SGLT-2 inhibitors primarily occur through the inhibition of the TGF-*β*/Smad signaling pathway and the suppression of CTGF expression. Studies have shown that SGLT-2 inhibitors reduce TGF-β activation, thereby inhibiting the downstream activation of the Smad signaling pathway (54). This reduces the synthesis of collagen and other matrix components, alleviating tubular-interstitial fibrosis. Furthermore, SGLT-2 inhibitors can lower the expression of CTGF, preventing the further amplification of fibrotic signaling and slowing the progression of fibrosis. Through these mechanisms, SGLT-2 inhibitors effectively reduce structural kidney damage, protect the renal tubulointerstitium, and prevent the worsening of fibrosis. Slowing the progression of renal fibrosis helps improve kidney function and delay the progression of CKD (55).

SGLT-2 inhibitors not only treat diabetes by lowering blood glucose but also play a significant role in CKD management through their dual anti-inflammatory and anti-fibrotic mechanisms. They reduce the production of pro-inflammatory cytokines, alleviating renal inflammation. At the same time, by inhibiting the activation of TGF-*β* and CTGF signaling pathways, they mitigate the fibrotic process in the renal tubulointerstitium (56). These mechanisms provide strong theoretical support for the application of SGLT-2 inhibitors in CKD and other related conditions.

### 2.4 Systemic metabolic improvement

SGLT-2 inhibitors not only treat diabetes but also improve systemic metabolism through multiple mechanisms. These effects, particularly in lowering blood pressure, uric acid, and body weight, are relevant to diabetes management and also indirectly benefit kidney function (57).

#### 2.4.1 Mechanism of blood pressure reduction

SGLT-2 inhibitors lower blood pressure through several mechanisms. First, by inhibiting the SGLT-2 protein in the renal proximal tubule, the reabsorption of glucose is reduced, promoting the excretion of both glucose and water. The loss of water along with glucose leads to a reduction in blood volume, which helps lower blood pressure (58). Additionally, SGLT-2 inhibitors reduce sodium reabsorption in the kidneys, increasing sodium excretion and lowering the body’s sodium load. This further decreases blood volume and extracellular fluid, contributing to blood pressure reduction. Furthermore, SGLT-2 inhibitors reduce sympathetic nervous system activation, which plays a crucial role in managing hypertension. The reduction in sympathetic activity further supports the decline in blood pressure (59).

#### 2.4.2 Mechanism of uric acid reduction

SGLT-2 inhibitors also play a key role in lowering serum uric acid levels. Normally, the kidneys reabsorb most of the filtered uric acid in the proximal tubule. By reducing glucose and sodium reabsorption, SGLT-2 inhibitors alter the kidney’s handling of uric acid (60). Specifically, SGLT-2 inhibitors inhibit uric acid reabsorption and increase its excretion, effectively lowering serum uric acid levels. This action is particularly beneficial for patients with hyperuricemia and gout. Additionally, by reducing the metabolic burden on the kidneys, SGLT-2 inhibitors indirectly improve tubular function, further reducing uric acid accumulation (61).

#### 2.4.3 Mechanism of weight reduction

SGLT-2 inhibitors reduce body weight by promoting the excretion of glucose and water, which decreases caloric intake and body water. The excretion of glucose not only lowers blood glucose levels but also removes associated energy, thus reducing overall calorie intake (62). Weight loss is typically accompanied by improvements in fat metabolism. SGLT-2 inhibitors encourage the body to rely more on fat as an energy source, further reducing body fat. Additionally, they may improve insulin sensitivity, helping reduce fat accumulation, particularly in type 2 diabetes patients, where improving insulin resistance is a critical factor in weight management (63).

## 3 Evaluation of efficacy

CKD not only has a profound impact on patients’ physiological functions but also significantly diminishes their quality of life. Symptoms such as fatigue, reduced mobility, sleep disturbances, and increased psychological stress often create numerous challenges in daily life (64). These issues not only limit patients’ daily activities and social interactions but can also lead to emotional distress and depression. In recent years, SGLT-2 inhibitors have emerged as a novel class of drugs that show not only significant efficacy in preserving kidney function and reducing cardiovascular risks but also hold potential in improving the quality of life of patients with CKD. By optimizing energy metabolism, reducing inflammation, and improving sleep quality, SGLT-2 inhibitors offer new possibilities for enhancing the overall quality of life for patients (65).

### 3.1 Key clinical trials

The efficacy of SGLT-2 inhibitors has been validated in several key clinical trials in recent years, particularly in studies such as DAPA-CKD, CREDENCE, and EMPA-KIDNEY. The following section provides a more detailed overview of these studies, their findings, and the implications for the use of SGLT-2 inhibitors in the treatment of CKD (66). The DAPA-CKD study was a large, multicenter, randomized, double-blind, placebo-controlled phase III clinical trial designed to evaluate the efficacy of Dapagliflozin in patients with CKD (67). The study design was crucial as it included both diabetic and non-diabetic patients, which enhances its clinical relevance and broadens its applicability. The results of the DAPA-CKD study indicated that Dapagliflozin significantly slowed the decline in estimated glomerular filtration rate (eGFR) in CKD patients (68). Specifically, Dapagliflozin effectively slowed tubulointerstitial damage and reduced injury to renal tubular epithelial cells, thereby delaying the progression of kidney dysfunction. The study data showed that the rate of eGFR decline in the Dapagliflozin treatment group was significantly slower than in the placebo group, indicating its positive role in kidney function preservation (69). The study also found that Dapagliflozin significantly reduced the incidence of ESRD. Patients with ESRD often require dialysis or kidney transplantation, and the use of Dapagliflozin effectively reduced the need for these treatments. Specifically, Dapagliflozin significantly reduced the risk of progression to ESRD, thereby delaying the need for such treatments (70).

In addition to kidney protection, Dapagliflozin also exhibited significant cardiovascular protective effects. The study showed that Dapagliflozin reduced the risk of cardiovascular death, hospitalization for heart failure, and other adverse cardiovascular events (71). The effect of Dapagliflozin was particularly pronounced in CKD patients with heart failure. Dapagliflozin improved cardiac structure and function, reducing the burden on the heart and consequently lowering the risk of cardiovascular events (72). A key feature of the DAPA-CKD study was that it included both diabetic and non-diabetic patients, making its findings more broadly applicable. The study found that Dapagliflozin showed almost no difference in efficacy for CKD, regardless of whether the patient had diabetes (73). This finding overcomes the previous limitation of SGLT-2 inhibitors being primarily used in diabetic patients, offering a new treatment option for other types of CKD patients. The DAPA-CKD study clarified the important role of Dapagliflozin in the treatment of CKD, particularly in delaying kidney failure, reducing ESRD incidence, and providing cardiovascular protection. The findings of the study provide strong evidence supporting the use of SGLT-2 inhibitors in non-diabetic CKD patients, promoting their widespread clinical application (74).

The CREDENCE study is a multicenter, randomized, double-blind, placebo-controlled phase III clinical trial targeting patients with type 2 diabetes. Its primary aim was to evaluate the efficacy and safety of Canagliflozin in patients with diabetes-related CKD (75). Although the study mainly focused on diabetic patients, its subgroup analyses provided valuable insights into the effects on non-diabetic patients as well. The CREDENCE study demonstrated that Canagliflozin significantly reduced the progression of kidney dysfunction in patients with diabetes-related CKD (76). The study indicated that Canagliflozin slowed the decline in eGFR and significantly reduced the proportion of patients requiring dialysis or kidney transplantation. The primary endpoint of the study was a composite kidney endpoint (including significant eGFR decline, death, or ESRD occurrence), with the Canagliflozin group showing significantly better results than the placebo group (77).

Canagliflozin also exhibited significant cardiovascular protective effects. The CREDENCE study found that Canagliflozin significantly reduced cardiovascular mortality, particularly showing a clear advantage in reducing hospitalizations for heart failure (78). This result further supports that SGLT-2 inhibitors not only improve kidney function but also provide significant benefits for cardiovascular health. Although the CREDENCE study mainly targeted diabetic patients, its subgroup analysis also showed significant kidney protective effects of Canagliflozin in non-diabetic patients. Non-diabetic CKD patients also benefited from Canagliflozin treatment, with slowed progression of kidney dysfunction and reduced risk of ESRD (14). The CREDENCE study further validated the kidney protective effect of Canagliflozin in diabetic patients, and confirmed that non-diabetic patients also benefit. This study not only supports the efficacy of SGLT-2 inhibitors in diabetes-related CKD but also lays the groundwork for their use in non-diabetic patients (79).

The ongoing EMPA-KIDNEY study is a key clinical trial aimed at further exploring the efficacy of Empagliflozin in patients with various types of CKD. The study includes both diabetic and non-diabetic patients and employs rigorous clinical design to assess the effects of Empagliflozin on kidney function protection and cardiovascular disease prevention (80). EMPA-KIDNEY is a multicenter, randomized, double-blind, placebo-controlled phase III clinical trial. Its primary endpoints include kidney function decline (e.g., significant eGFR reduction), the incidence of ESRD, and mortality. Secondary endpoints include cardiovascular death and hospitalizations for heart failure. The participants include patients with various types of CKD, both diabetic and non-diabetic (81).

While final data from the EMPA-KIDNEY study have not been fully published, preliminary results have already shown significant efficacy of Empagliflozin in patients with CKD. Preliminary results indicate that Empagliflozin significantly slowed the decline in eGFR and delayed the progression of kidney failure (80). Preliminary analysis suggests that Empagliflozin is particularly effective in diabetic patients but also demonstrates kidney-protective effects in non-diabetic patients. Early data show that Empagliflozin reduces cardiovascular mortality and hospitalizations for heart failure, further validating the role of SGLT-2 inhibitors in cardiovascular disease prevention. Preliminary data suggest that Empagliflozin also has kidney-protective effects in non-diabetic CKD patients, supporting its potential use in a broad patient population (82). The EMPA-KIDNEY study provides preliminary evidence for the application of Empagliflozin in CKD patients. Although final data have not been fully released, the existing preliminary results already show significant efficacy of Empagliflozin in both diabetic and non-diabetic patients, particularly in terms of kidney and cardiovascular protection (83).

The DAPA-CKD, CREDENCE, and EMPA-KIDNEY studies provide strong evidence for the efficacy of SGLT-2 inhibitors (such as Dapagliflozin, Canagliflozin, and Empagliflozin) in CKD. SGLT-2 inhibitors demonstrate significant benefits in several areas: slowing the decline in eGFR, delaying kidney failure, reducing the incidence of ESRD, lowering cardiovascular mortality and heart failure hospitalizations, and improving cardiovascular health (84). SGLT-2 inhibitors offer kidney protection in both diabetic and non-diabetic patients, providing treatment options for a broader patient population. These findings support the clinical application of SGLT-2 inhibitors in CKD, especially in both diabetic and non-diabetic patients, providing valuable data for optimizing future treatment strategies (85).

### 3.2 Kidney outcomes

The use of SGLT-2 inhibitors in diabetic and CKD patients not only improves metabolic status by lowering blood glucose but also enhances kidney function through various direct and indirect mechanisms. Their significant impact on kidney outcomes is primarily seen in slowing the decline in eGFR and reducing the incidence of proteinuria. The mechanisms behind these effects are complex, involving kidney hemodynamics, metabolic pathways, and oxidative stress responses (13).

The rate of decline in eGFR is a crucial indicator of kidney failure, and slowing this decline is vital. SGLT-2 inhibitors exert their effects through several key mechanisms. First, SGLT-2 inhibitors improve renal hemodynamics, particularly by influencing the kidney’s perception of sodium, enhancing the tubulo-glomerular feedback mechanism, reducing glomerular filtration pressure, and preventing glomerular hypertension-related damage, thus easing the burden on the glomeruli (86). Additionally, by reducing the reabsorption of glucose and sodium in the renal proximal tubules, SGLT-2 inhibitors reduce the kidney’s metabolic load, helping to slow kidney damage caused by excessive glucose and sodium reabsorption. SGLT-2 inhibitors also improve kidney metabolism, reducing oxidative stress and inflammation caused by hyperglycemia, thus reducing damage to the kidneys and effectively delaying the decline in eGFR. More importantly, SGLT-2 inhibitors help maintain kidney structure and function by reducing fibrosis, inhibiting apoptosis, and suppressing the release of fibrotic factors (87–89).

Proteinuria is an early marker of kidney damage and an important indicator of poor prognosis in CKD patients. The mechanism by which SGLT-2 inhibitors significantly reduce proteinuria mainly involves alleviating glomerular hyperfiltration, modulating tubular function, and reducing oxidative stress and inflammation. Diabetic and CKD patients often experience glomerular hyperfiltration (90). SGLT-2 inhibitors improve the tubulo-glomerular feedback mechanism, reduce pressure within the glomeruli, and prevent protein leakage, thus lowering proteinuria levels. By reducing glucose and sodium reabsorption in the renal tubules, SGLT-2 inhibitors alleviate the metabolic burden of the tubules, contributing to the restoration of normal renal blood flow and function, further reducing proteinuria (91).

SGLT-2 inhibitors also reduce renal tubular damage, oxidative stress, and inflammation, helping to decrease proteinuria induced by these factors. In a hyperglycemic environment, excessive glucose reabsorption increases the burden on the kidneys. SGLT-2 inhibitors reduce glucose reabsorption, indirectly decreasing proteinuria caused by hyperglycemia. The effects of SGLT-2 inhibitors are prominent not only in diabetic kidney disease patients but also in early- and mid-stage CKD patients, who can also benefit from their use (92). In early CKD patients, who have higher glomerular filtration rates and significant proteinuria, SGLT-2 inhibitors can significantly delay the decline in eGFR and reduce disease progression. In mid-stage CKD patients, although kidney function is partially impaired, SGLT-2 inhibitors continue to improve kidney outcomes through mechanisms such as enhancing renal metabolism, reducing oxidative stress and inflammation, and lowering proteinuria. Overall, SGLT-2 inhibitors slow the decline in kidney function, reduce proteinuria levels, and significantly improve kidney outcomes in patients with diabetic nephropathy and CKD (93).

**Figure 2:**
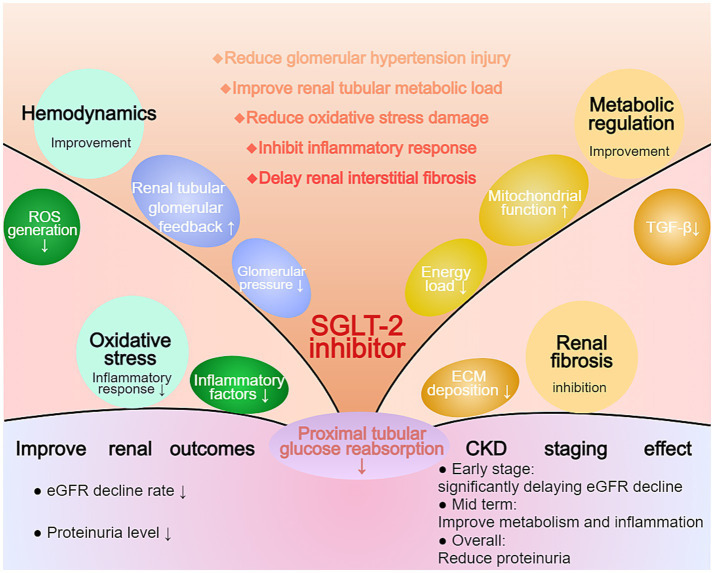
Multifaceted Effects of SGLT-2 Inhibition on Renal Protection in CKD. The diagram illustrates the comprehensive effects of SGLT-2 inhibitors on renal outcomes. The central mechanism involves reduced proximal tubular glucose reabsorption, leading to improvements in hemodynamics, oxidative stress, and metabolic regulation. Key outcomes include reduced eGFR decline rate and proteinuria levels. The CKD staging effect demonstrates significant benefits across different disease stages, particularly in early-stage CKD by delaying eGFR decline and improving metabolic parameters.

SGLT-2 inhibitors work through multiple pathways, achieving significant results in slowing eGFR decline and improving proteinuria. Their mechanisms include improving renal hemodynamics, reducing the kidney’s metabolic burden, decreasing oxidative stress and inflammation, and improving renal interstitial fibrosis. These mechanisms work together to reduce kidney damage in diabetic nephropathy and CKD patients, leading to improved kidney outcomes (Figure 2).

### 3.3 Cardiovascular outcomes

The use of SGLT-2 inhibitors, such as empagliflozin and dapagliflozin, in heart failure patients has been validated in multiple large-scale clinical trials. These trials demonstrate significant cardiovascular protective effects in patients with both preserved and reduced ejection fraction. The EMPEROR-Preserved and DELIVER studies show that empagliflozin and dapagliflozin significantly reduce cardiovascular mortality and hospitalization risk in heart failure patients with preserved ejection fraction (94). Additionally, these drugs improve quality of life, alleviate heart failure symptoms, and enhance exercise tolerance. These findings indicate that SGLT-2 inhibitors are clinically valuable not only for patients with reduced ejection fraction but also for those with preserved ejection fraction (95).

The EMPEROR-Reduced and DAPA-HF studies further confirm the efficacy of SGLT-2 inhibitors in chronic heart failure patients with reduced ejection fraction. In these trials, empagliflozin and dapagliflozin significantly reduced the risks of cardiovascular death, heart failure hospitalization, and all-cause mortality (96). Additionally, these drugs markedly improved patient symptoms and quality of life, highlighting their critical role in enhancing clinical outcomes in heart failure. These findings provide robust evidence supporting the inclusion of SGLT-2 inhibitors in standard heart failure treatment, particularly for patients with reduced ejection fraction (97).

The mechanism of action of SGLT-2 inhibitors may involve reducing sodium and water retention, alleviating cardiac workload, and improving left ventricular function. Additionally, these drugs may protect the cardiovascular system by decreasing inflammation and oxidative stress. Concurrently, SGLT-2 inhibitors exert a protective effect on the kidneys, further enhancing cardiovascular health, particularly in heart failure patients with renal impairment, where their efficacy is more pronounced (57).

In conclusion, results from clinical trials such as EMPEROR-Preserved, DELIVER, EMPEROR-Reduced, and DAPA-HF demonstrate that SGLT-2 inhibitors exert broad cardiovascular protective effects in heart failure treatment. These drugs significantly improve cardiovascular outcomes and enhance patients’ quality of life. Consequently, SGLT-2 inhibitors should be considered a cornerstone of heart failure therapy, offering substantial clinical value for patients with both preserved and reduced ejection fraction (96).

### 3.4 Quality of life

CKD patients often experience significant fatigue, which affects their daily quality of life and limits their physical activity and social interactions. SGLT-2 inhibitors alleviate fatigue through various mechanisms, including optimizing energy metabolism, reducing inflammation, and improving sleep quality (98). Specifically, SGLT-2 inhibitors promote fatty acid oxidation, providing a more sustained and efficient energy source, reducing dependence on glucose, and preventing energy supply instability. CKD is often associated with low-grade systemic inflammation. SGLT-2 inhibitors have anti-inflammatory effects, reducing the levels of inflammatory markers and alleviating fatigue. Additionally, by reducing nocturnal urination, SGLT-2 inhibitors improve sleep quality, which in turn reduces daytime fatigue (99).

Improved exercise tolerance allows patients to engage in daily activities for longer periods, enhancing self-care abilities and independence. SGLT-2 inhibitors enhance physical endurance by improving cardiac function, reducing anemia, and optimizing fluid balance (100). Specifically, SGLT-2 inhibitors improve heart function, reduce cardiac workload, and decrease the incidence of heart failure, allowing patients to feel more energetic during physical activities. CKD is often accompanied by anemia. SGLT-2 inhibitors stimulate erythropoietin production, improving anemia and increasing physical strength and endurance (101). At the same time, through diuresis, SGLT-2 inhibitors help regulate fluid balance, reducing edema and discomfort, making it easier for patients to engage in physical activities. SGLT-2 inhibitors improve quality of life in non-diabetic CKD patients through various physiological and metabolic mechanisms. First, in terms of renal protection, SGLT-2 inhibitors inhibit glomerular hyperfiltration by increasing glomerular inflow, reducing kidney pressure, delaying kidney function deterioration, and decreasing urinary protein excretion, thereby reducing kidney damage (102). In terms of metabolic effects, SGLT-2 inhibitors promote fatty acid oxidation and ketone body production, providing more efficient energy sources that help increase stamina and reduce fatigue. They also promote the excretion of glucose and fats, helping with weight control, reducing weight-related burdens, and enhancing physical activity (103).

In terms of cardiovascular effects, SGLT-2 inhibitors have a mild blood pressure-lowering effect by reducing fluid volume and dilating blood vessels, which helps reduce cardiac workload and improve heart function. Furthermore, long-term use of SGLT-2 inhibitors reduces cardiac hypertrophy and fibrosis, improving heart structure and function and enhancing endurance and physical strength (58). Other systemic effects include anti-inflammatory and antioxidant actions. SGLT-2 inhibitors reduce oxidative stress and inflammation, which is particularly important for CKD patients and helps improve overall health and quality of life. Simultaneously, through their diuretic effects, SGLT-2 inhibitors help maintain the balance of electrolytes like sodium and potassium, reducing muscle cramps and fatigue (51).

Multiple clinical trials and observational studies have confirmed that SGLT-2 inhibitors not only delay the decline of kidney function in non-diabetic CKD patients but also significantly improve their quality of life. For example, the DAPA-CKD trial showed that patients treated with Dapagliflozin had significant improvements in physical activity, fatigue levels, and overall quality of life scores (104).

SGLT-2 inhibitors, through various physiological and metabolic mechanisms, not only significantly slow the progression of non-diabetic CKD but also improve fatigue and enhance physical endurance, thereby significantly improving patients’ overall quality of life. This makes SGLT-2 inhibitors an important therapeutic option for non-diabetic CKD, addressing not only kidney function protection but also the patient’s daily life and mental well-being (95).

### 3.5 Safety evaluation

Although SGLT-2 inhibitors have shown significant therapeutic benefits in CKD patients, their use requires close monitoring of potential side effects and safety concerns. This section will explore common adverse effects of SGLT-2 inhibitors, particularly urinary tract infections, hypotension, and diabetic ketoacidosis, while analyzing the specific needs and risk factors of different populations. Additionally, the long-term safety of these medications will be assessed to ensure that clinical use balances efficacy with risk, maximizing patient health and quality of life. A comprehensive safety evaluation will enable healthcare professionals to develop more effective personalized treatment plans, optimize the use of SGLT-2 inhibitors, and improve the overall prognosis for non-diabetic CKD patients (22).

#### 3.5.1 Common adverse effects

However, attention must be paid to common adverse effects during use. One of the most frequent side effects is urinary tract infections. These drugs inhibit the SGLT-2 protein in the kidneys, reducing glucose reabsorption and leading to increased glucose excretion in urine (105). This high-glucose environment provides ideal conditions for bacterial and fungal growth, particularly in female patients, which may result in vaginal candidiasis or urinary tract infections. Another concern is the diuretic effect of SGLT-2 inhibitors, which can lead to fluid loss and result in hypotension. This effect is especially pronounced in elderly patients or those on other antihypertensive medications (106). Fluid loss reduces blood volume, leading to lower blood pressure, which may manifest as dizziness, fatigue, or even falls. In severe cases, it may cause insufficient organ perfusion, affecting overall health. Additionally, while less common, diabetic ketoacidosis (DKA) is an important side effect to consider. By promoting glucose excretion and lowering blood sugar levels, SGLT-2 inhibitors may reduce insulin requirements and increase glucagon secretion, leading to an increase in fatty acid breakdown and ketone production (107). This hormonal change may cause ketone accumulation, leading to acidosis. High-risk groups, such as insulin users, those with prolonged fasting or strict dietary control, and patients with acute illness, should be closely monitored for symptoms and treated promptly. In non-diabetic CKD patients, healthcare professionals should carefully monitor urinary tract health, blood pressure, and metabolic status, especially for high-risk individuals, to maximize therapeutic benefits while minimizing potential adverse effects (108).

#### 3.5.2 Considerations for special populations

SGLT-2 inhibitors have shown significant efficacy in treating non-diabetic CKD, particularly in slowing kidney function decline and offering cardiovascular protection. However, their use is associated with several common adverse effects that require careful attention from clinicians and patients. The most common and concerning side effects include urinary tract infections, hypotension, and DKA. Urinary tract infections are among the most common adverse effects in patients using SGLT-2 inhibitors (109). These medications work by inhibiting the SGLT-2 protein in the kidneys, reducing glucose reabsorption and leading to higher glucose concentrations in the urine. The resulting high-glucose environment creates ideal conditions for bacterial and fungal growth, particularly in female patients, potentially causing infections such as vaginal candidiasis, urethritis, or cystitis. Although less common in male patients, the risk still exists (110). Preventative measures include maintaining good personal hygiene, keeping the urinary tract clean, and seeking medical attention promptly if infection symptoms arise. Regular monitoring of urinary tract health is essential to identify and manage infections early, thereby reducing the risk of complications. Another concern is the diuretic effect of SGLT-2 inhibitors, which can lead to fluid loss and cause hypotension. By inhibiting sodium and glucose reabsorption, these medications increase the excretion of sodium and glucose, resulting in mild diuresis. This fluid loss can decrease blood volume, leading to a reduction in blood pressure (111). Hypotension is particularly common in elderly patients or those using other antihypertensive medications (e.g., diuretics, beta-blockers, ACE inhibitors). Symptoms of hypotension include dizziness, fatigue, orthostatic hypotension, and even falls. In severe cases, it may lead to insufficient organ perfusion, affecting the heart, brain, and kidneys. To prevent hypotension, it is recommended to gradually adjust the drug dosage at the start of treatment, closely monitor blood pressure, and modify the use of diuretics or other antihypertensive medications based on the patient’s individual needs (112). Although rare, DKA is another important side effect that warrants attention when using SGLT-2 inhibitors. These medications promote glucose excretion and lower blood sugar levels, which may reduce insulin requirements and increase glucagon secretion. This hormonal change stimulates fatty acid breakdown and ketone production, which can lead to ketone accumulation and acidosis. The symptoms of DKA are often non-specific, including nausea, vomiting, abdominal pain, fatigue, rapid breathing, and altered mental status, making it easy to overlook or misdiagnose. High-risk groups, such as insulin users, those on prolonged fasting or strict dietary control, patients with acute illnesses, and those with pancreatic dysfunction, should be particularly cautious. Preventative measures include temporarily discontinuing SGLT-2 inhibitors in high-risk situations, closely monitoring blood glucose and ketone levels, and providing timely medical intervention if symptoms arise (113).

While SGLT-2 inhibitors offer significant benefits in treating non-diabetic CKD, their potential adverse effects should not be overlooked. In clinical practice, healthcare professionals should consider each patient’s unique circumstances, balancing the therapeutic benefits with potential risks to develop personalized treatment plans (109). For patients prone to urinary tract infections, at risk of hypotension, or susceptible to diabetic ketoacidosis, enhanced monitoring and management are essential. Providing relevant health education and ensuring that the benefits of treatment are maximized while minimizing adverse effects will help protect patients’ health and quality of life (114).

#### 3.5.3 Long-term safety

When using SGLT-2 inhibitors for the long-term treatment of non-diabetic CKD, several safety considerations must be addressed. First, by increasing urinary glucose excretion, SGLT-2 inhibitors may lead to electrolyte imbalances, such as disturbances in sodium and potassium levels. Although current studies suggest good overall tolerance, the long-term risk of severe electrolyte imbalances remains unclear, and further long-term data are needed to assess this risk (115). Additionally, although SGLT-2 inhibitors may cause a temporary reduction in GFR in the early stages, it remains unclear whether long-term use has a cumulative impact on kidney function, and more research is needed to confirm this. The risk of infection is another important concern. The mechanism of these drugs increases urinary glucose, which may raise the risk of urinary tract infections and genital fungal infections. It is unclear whether long-term use will increase the severity or frequency of these infections, requiring further investigation. Although the incidence of DKA is low in non-diabetic patients, the potential for increased risk with long-term use still requires observation. Furthermore, some studies suggest that SGLT-2 inhibitors may affect bone metabolism, and whether long-term use increases the risk of fractures remains to be supported by further data (116).

SGLT-2 inhibitors work by inhibiting the SGLT-2 protein in the kidneys, reducing glucose reabsorption and increasing urinary glucose excretion, which lowers blood glucose levels. In non-diabetic patients, the renal protective effects of these drugs are primarily achieved by reducing kidney workload—by lowering GFR and alleviating the high-pressure state in the renal interstitium, thereby slowing the progression of kidney damage (117). Additionally, SGLT-2 inhibitors have diuretic and antihypertensive effects, which help reduce blood pressure and alleviate cardiovascular strain, contributing to kidney protection. Moreover, these drugs may reduce kidney inflammation and fibrosis by modulating cellular metabolism and reducing oxidative stress, further enhancing renal protection. However, the cumulative effects of these mechanisms with long-term use remain to be fully studied. In particular, the metabolic pathways and physiological impacts of the drug may vary in non-diabetic populations, necessitating more research to comprehensively understand its long-term safety and efficacy (118).

### 3.6 Effects on other subtypes of kidney disease

Initially developed for type 2 diabetes, SGLT-2 inhibitors have demonstrated significant efficacy in various kidney diseases, extending beyond their original indications. These drugs protect the kidneys and slow renal function decline by improving renal hemodynamics, reducing glomerular pressure, decreasing proteinuria, and mitigating inflammation and fibrosis. Consequently, they show promise in conditions such as IgA nephropathy, autosomal dominant polycystic kidney disease (ADPKD), and focal segmental glomerulosclerosis (119). Given their multi-target effects, SGLT-2 inhibitors are emerging as a novel therapeutic option for diverse chronic kidney diseases. Non-diabetic CKD, including ADPKD, glomerulonephritis, and hypertension-induced kidney damage, arises from distinct etiologies and mechanisms, necessitating tailored treatment approaches. In ADPKD, a genetic disorder, therapy focuses on slowing cyst growth and delaying renal failure. Glomerulonephritis, driven by immune dysregulation, often requires immunosuppressive therapy to control inflammation. Hypertension-induced kidney damage emphasizes blood pressure management to mitigate renal injury (13). Treatment options for non-diabetic CKD remain limited, highlighting the urgent need for innovative strategies. In this context, SGLT-2 inhibitors offer substantial potential as a new therapeutic avenue for non-diabetic CKD. By alleviating tubular pressure, reducing proteinuria, and suppressing inflammation and fibrosis, these drugs significantly slow disease progression and improve patient outcomes. The exploration and application of this approach provide renewed hope for individuals with non-diabetic CKD (109).

IgA nephropathy is characterized by the deposition of immune complexes in the mesangium, leading to localized inflammation and mesangial proliferation, resulting in glomerular damage, proteinuria, and gradual kidney function deterioration. SGLT-2 inhibitors inhibit glucose and sodium reabsorption in the proximal tubules, allowing more sodium to reach the distal nephron and activating the tubuloglomerular feedback mechanism (120). This process causes afferent arteriole constriction, reducing glomerular pressure and filtration rate, thereby minimizing mechanical stress damage to the glomeruli. Additionally, reducing the hyperfiltration state within the kidney decreases protein leakage, mitigating proteinuria, which not only serves as a marker but also interrupts the vicious cycle that promotes interstitial fibrosis. Furthermore, SGLT-2 inhibitors improve metabolic status, reduce body weight, control blood pressure, and improve lipid profiles, indirectly alleviating chronic inflammation and fibrosis, which ultimately benefits the overall renal prognosis in IgA nephropathy patients (121).

ADPKD is caused by mutations in the PKD1 and PKD2 genes, leading to the progressive enlargement of multiple kidney cysts and a decline in kidney function. SGLT-2 inhibitors reduce sodium reabsorption in the proximal tubules, activating the TGF mechanism to lower glomerular filtration rate and filtration pressure, relieving local high-pressure states, reducing kidney unit damage and compensatory hypertrophy, which may slow the progression of renal parenchymal damage and cyst expansion (122). Moreover, SGLT-2 inhibitors improve overall metabolic and hemodynamic conditions, control blood pressure, reduce fluid overload, and decrease excessive RAAS activation and other signaling pathways that promote cyst growth. Although the precise mechanisms still require further validation, these effects provide a theoretical basis for the use of SGLT-2 inhibitors in ADPKD (58).

Focal Segmental Glomerulosclerosis (FSGS) is a disease characterized by localized and partial sclerosis of the glomeruli, often resulting in proteinuria and gradual decline in kidney function. SGLT-2 inhibitors reduce glomerular filtration rate, decrease capillary pressure in the glomeruli, and lower protein leakage, thereby alleviating mechanical stress on the glomeruli and blocking the fibrotic process induced by proteinuria (123). Additionally, they modulate glomerular hemodynamics through the TGF mechanism, mitigating the hyperfiltration state and reducing the chronic inflammatory and fibrotic processes associated with FSGS. Preliminary clinical data suggest that SGLT-2 inhibitors have the potential to reduce proteinuria and delay the progression of kidney dysfunction. Further randomized controlled trials are needed to confirm their efficacy and safety (124).

Lupus nephritis is a severe complication of systemic lupus erythematosus, characterized by the deposition of immune complexes in the glomeruli, leading to inflammation and kidney dysfunction. SGLT-2 inhibitors reduce glomerular filtration rate and decrease proteinuria, alleviating mechanical stress on the glomeruli and the protein-induced kidney damage (125). Moreover, SGLT-2 inhibitors may reduce the secretion of inflammatory cytokines and inhibit the infiltration of inflammatory cells, thereby reducing kidney tissue inflammation. By improving glucose control, lowering blood pressure, and reducing fluid overload, SGLT-2 inhibitors help create a more favorable renal environment, slowing the progression of kidney dysfunction. Although specific studies on lupus nephritis are limited, the known anti-inflammatory and kidney-protective mechanisms provide a theoretical basis for their use in this condition. More targeted clinical trials are needed to evaluate their real-world efficacy and safety (51).

Membranous nephropathy is a common glomerular disease in adults, characterized by the deposition of immune complexes in the glomerular basement membrane, leading to proteinuria and kidney function deterioration. SGLT-2 inhibitors reduce glomerular filtration rate and decrease intraglomerular pressure, effectively lowering proteinuria levels and alleviating mechanical damage to the glomeruli. Long-term use of SGLT-2 inhibitors may help reduce pathological changes in the glomerular basement membrane, preventing or delaying glomerulosclerosis (126). Furthermore, by reducing proteinuria and improving metabolic status, SGLT-2 inhibitors may indirectly suppress fibrosis and inflammation, protecting kidney structure. Although preliminary studies on membranous nephropathy show potential for reducing proteinuria and protecting kidney function, more clinical data are required to confirm their specific effects and the optimal usage strategy (38).

Chronic tubulointerstitial kidney disease includes various conditions that cause chronic kidney failure due to damage to the tubules and interstitial tissue. SGLT-2 inhibitors reduce glucose and sodium reabsorption, decreasing the workload of the renal tubules and alleviating metabolic stress (127). They may also reduce the infiltration of inflammatory cells and inhibit fibrosis signaling pathways, slowing the progression of interstitial fibrosis. By optimizing renal hemodynamics, SGLT-2 inhibitors help maintain the nutritional supply and function of the interstitial tissue. Given the limited treatment options for chronic tubulointerstitial kidney disease, SGLT-2 inhibitors provide a promising new avenue for treatment. Further research will help clarify their specific value in treating these conditions (128).

Additionally, SGLT-2 inhibitors protect against hypertensive kidney disease by lowering blood pressure and improving renal hemodynamics. In hereditary kidney diseases such as Alport syndrome, SGLT-2 inhibitors reduce glomerular pressure and proteinuria, thereby delaying kidney failure. Patients with CKD and heart disease may benefit from the renal and cardiac protective effects of SGLT-2 inhibitors (14). In patients with CKD and anemia, improved kidney function may indirectly alleviate anemia caused by renal failure. Overall, SGLT-2 inhibitors demonstrate significant renal protection across a variety of CKDs by lowering glomerular filtration rate, reducing proteinuria, improving blood pressure and metabolic status, and possessing anti-inflammatory and anti-fibrotic effects. Although the specific efficacy and safety in different kidney disease subtypes still require further clinical trials, current evidence supports SGLT-2 inhibitors as a multi-target renal protective agent with broad potential applications in the management of CKD (129).

### 3.7 Combination with other renal protective drugs

Combination therapies involving SGLT-2 inhibitors exhibit significant synergistic effects, particularly when paired with novel mineralocorticoid receptor antagonists (NS-MRAs), aldosterone synthase inhibitors (ASIs), AT1 blockers, or anti-TGF-*β* antibodies. These combinations enhance renal protection and slow CKD progression through complementary mechanisms, improving patient outcomes (130).

NS-MRAs, such as amiloride and eplerenone, inhibit mineralocorticoid receptors, reducing sodium retention, blood pressure, and renal fibrosis. When combined with SGLT2 inhibitors, which improve renal metabolism and hemodynamics, decrease oxidative stress and proteinuria, and activate tubuloglomerular feedback, these drugs complement NS-MRAs’ antifibrotic effects. This synergy alleviates renal inflammation, proteinuria, and fibrosis, reducing kidney workload and slowing functional decline (131).

ASIs, such as spironolactone, suppress overactive mineralocorticoid receptors, mitigating sodium retention, hypertension, and inflammation while inhibiting fibrosis. Paired with SGLT2 inhibitors, which regulate renal metabolism and hemodynamics and reduce tubular stress via enhanced glucose and sodium excretion, ASIs further suppress fibrotic factor release. Together, they reduce proteinuria, inflammation, and oxidative damage, delaying CKD progression (132).

AT1 blockers, such as losartan and irbesartan, inhibit angiotensin II, lowering glomerular pressure, tubular damage, and fibrosis while improving renal blood flow. Combined with SGLT2 inhibitors, which reduce local glomerular pressure, oxidative stress, and inflammation, this pairing synergistically mitigates tubular stress, proteinuria, and fibrosis, offering robust renal protection (133).Anti-TGF-*β* antibodies directly inhibit the TGF-β pathway, a key driver of renal fibrosis, reducing extracellular matrix deposition and fibroblast proliferation. SGLT2 inhibitors complement this by decreasing oxidative stress and inflammation, indirectly suppressing TGF-*β* activation. This dual approach—SGLT2 inhibitors improving metabolism and reducing renal burden, and anti-TGF-β antibodies blocking TGF-β signaling—markedly enhances antifibrotic effects, preserving renal structure and function (134).

Overall, combining SGLT2 inhibitors with NS-MRAs, ASIs, AT1 blockers, or anti-TGF-β antibodies leverages multiple mechanisms to enhance renal protection. These therapies reduce proteinuria, improve kidney function, and slow fibrosis while alleviating renal burden, inflammation, and oxidative stress. Future research should optimize these combinations and their timing to maximize renal benefits and improve clinical outcomes (135).

## 4 Potential effects of SGLT-2 inhibitors on non-renal systems

SGLT-2 inhibitors have demonstrated significant efficacy in renal protection, and their multifaceted effects on the cardiovascular and metabolic systems are equally important. Recent clinical studies have shown that SGLT-2 inhibitors significantly reduce the hospitalization rates for heart failure and the risk of cardiovascular death in CKD patients, while effectively managing metabolic diseases like obesity and hyperuricemia (136). These dual protective effects not only expand the clinical indications for SGLT-2 inhibitors but also offer new strategies and hope for the comprehensive treatment of non-diabetic CKD patients. The following sections will explore in detail the mechanisms of cardiovascular protection and metabolic disease regulation by SGLT-2 inhibitors and their clinical significance (13, 137–139).

### 4.1 Cardiovascular protection

Recent clinical studies have shown that SGLT-2 inhibitors also have significant cardiovascular protective effects in non-diabetic CKD patients, especially in reducing the hospitalization rates for heart failure and the risk of cardiovascular death. These benefits not only broaden the indications for SGLT-2 inhibitors but also provide new strategies for the comprehensive treatment of non-diabetic CKD patients (140). In large clinical trials such as DAPA-CKD and EMPA-KIDNEY, SGLT-2 inhibitors significantly reduced heart failure-related hospitalization rates and the risk of cardiovascular death in non-diabetic CKD patients. These trial results not only confirm the efficacy of SGLT-2 inhibitors in reducing cardiovascular events but also highlight their potential in improving overall cardiovascular health. Specifically, SGLT-2 inhibitors exert their protective effects through various mechanisms, including improving vascular function and optimizing cardiac metabolism (141). First, SGLT-2 inhibitors significantly improve vascular function. They reduce blood volume and lower blood pressure, alleviating both the preload and afterload on the heart, thereby reducing cardiac workload. Additionally, SGLT-2 inhibitors inhibit sympathetic nervous activity, reduce vascular resistance, and promote vasodilation. This vasodilation helps reduce arterial stiffness and improve vascular elasticity, further alleviating the strain on the heart. CKD patients often experience vascular dysfunction and arteriosclerosis. SGLT-2 inhibitors improve these pathological changes, protect vascular health, and reduce the occurrence of cardiovascular events. Secondly, SGLT-2 inhibitors play a key role in improving cardiac metabolic state. These drugs promote the remodeling of cardiac energy metabolism, shifting the heart muscle from glucose dependence to utilizing fatty acids and ketone bodies as primary energy sources. Ketone bodies, being more efficient energy carriers, improve myocardial energy efficiency, enhance myocardial contractility, and improve overall heart function (142, 143).

Additionally, SGLT-2 inhibitors inhibit the sodium-hydrogen exchanger (NHE), reducing intracellular sodium and calcium accumulation in myocardial cells, thereby alleviating myocardial fibrosis and improving cardiac structure and function. This mechanism helps reduce the occurrence of heart failure and delays pathological changes in cardiac structure (144). Furthermore, the diuretic and natriuretic effects of SGLT-2 inhibitors play a crucial role in reducing cardiac pressure and volume load. By promoting the excretion of sodium and water, SGLT-2 inhibitors effectively reduce the preload and afterload on the heart, decreasing cardiac pressure and volume load. This is significant for preventing and slowing the progression of heart failure. CKD patients often have fluid retention and hypertension. SGLT-2 inhibitors regulate fluid balance and improve blood pressure control, indirectly protecting heart function (118).

In addition to the main mechanisms, SGLT-2 inhibitors also protect cardiovascular health through other pathways. For example, these drugs lower uric acid levels, which are closely linked to an increased risk of cardiovascular disease. By promoting uric acid excretion, SGLT-2 inhibitors help reduce the occurrence of cardiovascular events (145). Moreover, SGLT-2 inhibitors improve kidney function, reduce glomerular pressure, and decrease renal fibrosis and inflammation, thereby delaying the decline of renal function. Kidney health is closely linked to heart function, and improving kidney function significantly reduces the strain on the heart, further protecting cardiovascular health. These cardiovascular protective effects are primarily attributed to the multiple regulatory actions of SGLT-2 inhibitors on the kidney-heart axis. There is a complex interaction between CKD and cardiovascular disease. Deteriorating kidney function often leads to hypertension, electrolyte imbalances, and fluid retention, which together increase the burden on the heart (74, 88).

**Figure 3:**
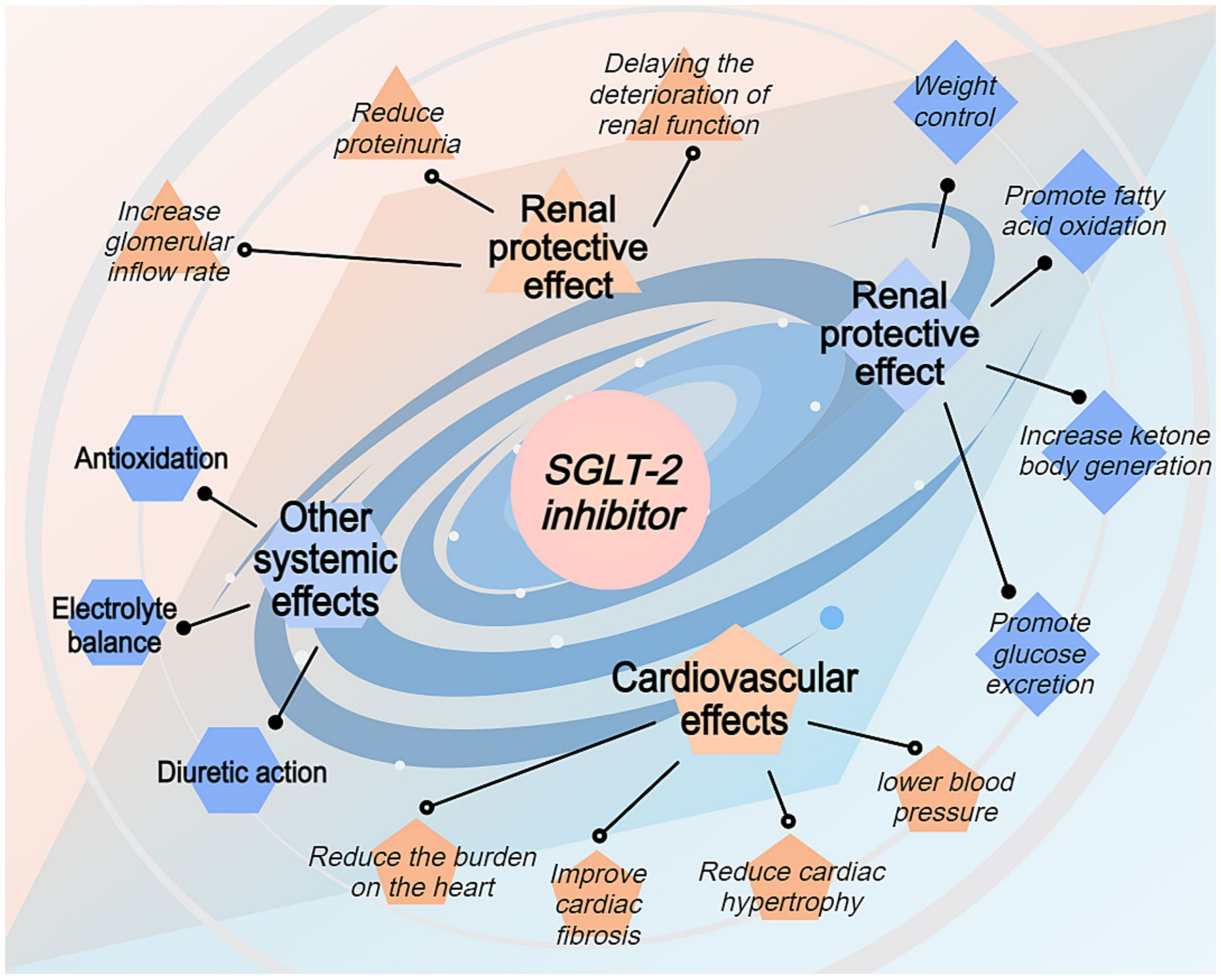
Systemic benefits of SGLT-2 inhibitors beyond renal protection. This figure depicts the broader therapeutic effects of SGLT-2 inhibitors through four main pathways: renal protective effects, cardiovascular effects, metabolic effects, and other systemic effects. The renal protective effects include reduced proteinuria and delayed renal function deterioration. Cardiovascular benefits encompass reduced cardiac hypertrophy, improved cardiac fibrosis, and lowered blood pressure. Metabolic effects include promoting fatty acid oxidation, increasing ketone body production, promoting glucose excretion, and weight control. Additional systemic effects include weight control, enhanced fatty acid oxidation, improved electrolyte balance, and antioxidation properties. The spiral design emphasizes the interconnected nature of these therapeutic benefits.

SGLT-2 inhibitors improve kidney function, lower blood pressure, and regulate fluid balance, thereby indirectly improving heart function. Additionally, SGLT-2 inhibitors enhance cardiovascular health by integrating multiple mechanisms, such as improving metabolic status and reducing inflammation and oxidative stress. The heart requires flexible energy metabolism to cope with stress and injury. SGLT-2 inhibitors promote more efficient energy use, enhancing myocardial metabolic flexibility, improving the heart’s ability to adapt to stress, and reducing the incidence of heart failure (Figure 3).

### 4.2 Regulation of metabolic diseases

In regulating obesity, SGLT-2 inhibitors work through multiple mechanisms. First, the drug inhibits the function of the SGLT-2 protein, reducing glucose reabsorption in the kidneys, leading to increased glucose excretion in the urine. This process not only directly reduces the body’s glucose load but also promotes the consumption of stored energy through the loss of calories, thereby aiding weight reduction. Additionally, the increased urinary glucose excretion induced by SGLT-2 inhibitors creates a mild energy deficit, activating fat oxidation pathways and reducing fat storage (135). Furthermore, SGLT-2 inhibitors may regulate hormones involved in fat metabolism, such as adiponectin and leptin, improving adipose tissue function and reducing chronic inflammation, which helps decrease lipogenesis and promote lipolysis. Moreover, the mild dehydration and electrolyte changes induced by the drug may stimulate the sympathetic nervous system, further increasing the basal metabolic rate and energy expenditure. These combined effects not only help reduce weight but also improve other components of metabolic syndrome, such as insulin sensitivity, blood pressure, and lipid levels, thereby lowering the risk of cardiovascular and renal diseases (146).

In improving hyperuricemia, SGLT-2 inhibitors also demonstrate significant effects. By inhibiting the SGLT-2 protein and increasing the excretion of sodium and glucose in the urine, the drug indirectly affects uric acid metabolism. Specifically, the increased excretion of sodium may inhibit uric acid reabsorption in the renal tubules, as there is a degree of synergy between the reabsorption of sodium and uric acid (147). Additionally, SGLT-2 inhibitors may directly interfere with uric acid transporters (e.g., URAT1), further reducing uric acid reabsorption and promoting its excretion. These mechanisms work together to significantly lower serum uric acid levels, reduce the frequency of gout attacks, and decrease uric acid deposition in the kidneys, preventing or delaying the progression of uric acid-induced kidney disease. On the other hand, the mild acidosis induced by the drug may alter the urinary pH balance, increasing the solubility of uric acid and promoting its excretion. Moreover, weight loss and improvement in metabolic syndrome also help reduce uric acid production and enhance its excretion efficiency. These multiple mechanisms give SGLT-2 inhibitors a significant advantage in controlling hyperuricemia, especially in non-diabetic CKD patients (60).

SGLT-2 inhibitors effectively regulate metabolic diseases such as obesity and hyperuricemia in non-diabetic CKD patients. By increasing the urinary excretion of glucose and sodium, these drugs not only promote weight loss and improve fat metabolism but also enhance uric acid excretion through multiple pathways, lowering blood uric acid levels. These combined effects not only improve the patient’s metabolic state but may also slow the progression of CKD and reduce the risk of cardiovascular complications, demonstrating the vast potential of SGLT-2 inhibitors in managing metabolic diseases.

## 5 Future outlook and challenges

SGLT-2 inhibitors, a novel class of glucose-lowering drugs, have shown significant efficacy and potential in treating non-diabetic CKD in recent years. While their use in diabetic nephropathy is widely recognized, their application in non-diabetic CKD patients is still in the exploratory stage.

### 5.1 In-depth mechanistic research

A deeper understanding of the differences in SGLT-2 inhibitors’ effects across various signaling pathways and their potential targets is key to advancing their application in non-diabetic CKD treatment. SGLT-2 inhibitors lower blood glucose levels by inhibiting the SGLT-2 protein in the renal proximal tubules, reducing glucose reabsorption. However, their renal protective effects extend beyond glucose-lowering and involve various complex biological mechanisms. First, SGLT-2 inhibitors reduce the kidney’s hyperfiltration state (13). By inhibiting sodium-glucose cotransport and promoting sodium excretion, they regulate glomerular hemodynamics, reduce glomerular filtration rate, and decrease glomerular pressure, thereby delaying further kidney function deterioration. Secondly, SGLT-2 inhibitors have significant anti-inflammatory and antifibrotic effects. Studies show that these drugs modulate inflammatory mediators like NF-κB and TGF-*β* signaling pathways, reducing inflammation and fibrosis in kidney tissues, thereby protecting renal structure and function (148, 149). Additionally, SGLT-2 inhibitors reduce ROS generation, suppress oxidative stress, and protect renal cells from oxidative damage. The synergistic action of these multiple mechanisms gives SGLT-2 inhibitors a unique advantage in renal protection (150–152). Future research needs to further elucidate the specific mechanisms of these signaling pathways, explore the differential effects of SGLT-2 inhibitors in various pathological states, and identify new potential therapeutic targets to optimize drug application strategies and enhance treatment efficacy (153–155).

### 5.2 Long-term research

Long-term efficacy studies in different populations and patients with specific kidney diseases are essential prerequisites for the application of SGLT-2 inhibitors in non-diabetic CKD treatment. While numerous studies have confirmed the efficacy of SGLT-2 inhibitors in adult diabetic patients, data on their long-term safety and effectiveness in non-diabetic CKD patients remain limited. First, research in pediatric patients is especially necessary (156). The pathological mechanisms of kidney diseases in children differ significantly from adults, and drug metabolism and responses vary as well. Long-term follow-up studies are needed to assess the safety, tolerance, and renal protective effects of SGLT-2 inhibitors in children, ensuring their safe use in this special population (157). Secondly, elderly patients, as a high-risk group for CKD, often have multiple chronic diseases and take several medications, so drug tolerance and potential drug interactions need special attention. Long-term studies should focus on assessing the risk–benefit balance of SGLT-2 inhibitors in elderly CKD patients to ensure their safe use in this population. Additionally, studying the efficacy and mechanisms of SGLT-2 inhibitors in patients with specific kidney diseases, such as polycystic kidney disease and autoimmune nephritis, is an important direction for future research (158). Through these long-term studies, we can comprehensively understand the application potential and possible risks of SGLT-2 inhibitors in different populations, providing stronger scientific evidence for clinical practice (159, 160).

### 5.3 Development of new indications

The multi-system effects of SGLT-2 inhibitors provide a broad outlook for their application in other chronic diseases. In addition to their use in kidney protection and cardiovascular diseases, research suggests that SGLT-2 inhibitors may show potential therapeutic value in neurodegenerative diseases and liver diseases. In addition to their use in kidney protection and cardiovascular diseases, research suggests that SGLT-2 inhibitors may show potential therapeutic value in neurodegenerative diseases and liver diseases (136). Specifically, SGLT-2 inhibitors can reduce oxidative stress and inflammation in neuronal cells, improve mitochondrial function, and enhance energy metabolism, thereby slowing the progression of neurodegenerative diseases. In liver diseases, non-alcoholic fatty liver disease (NAFLD) is closely related to metabolic syndrome. SGLT-2 inhibitors may positively affect NAFLD and its progression to liver fibrosis and cirrhosis by improving insulin resistance and reducing hepatic fat deposition (161). Additionally, existing studies have shown that SGLT-2 inhibitors offer good protection against heart failure and atherosclerosis, and their potential for use in other cardiovascular diseases can be further explored. Developing new indications not only expands the range of applications for SGLT-2 inhibitors but also brings clinical benefits to more patients. However, developing new indications requires rigorous clinical trials to verify their safety and efficacy in various diseases, ensuring that their use in new fields is based on solid scientific evidence and clinical value (162).

### 5.4 Economic and social impact

The widespread use of SGLT-2 inhibitors involves not only clinical efficacy but also various economic and social factors. First, cost-effectiveness analysis is crucial for evaluating the adoption of SGLT-2 inhibitors. Although these drugs are relatively expensive, their effectiveness in preventing kidney disease progression and reducing cardiovascular events could significantly lower overall healthcare costs in the long run. A detailed cost-effectiveness analysis helps policymakers devise appropriate strategies for promoting these drugs, supporting their integration into public health policies. Next, global implementation strategies must account for differences in healthcare systems, drug regulatory policies, and economic conditions across countries and regions (163). For instance, low-income countries may face challenges such as high drug prices and limited healthcare resources, requiring international cooperation and drug price negotiations to improve accessibility. Additionally, adjustments to insurance coverage and reimbursement policies are key factors in promoting the widespread use of SGLT-2 inhibitors. Including SGLT-2 inhibitors in insurance coverage can effectively reduce the financial burden on patients and increase drug utilization rates. To achieve this, close collaboration with national insurance agencies is required, providing sufficient clinical data to support reimbursement inclusion and ensure affordability for patients (164). Finally, social awareness and education are foundational to achieving economic and social benefits. Enhancing awareness among healthcare professionals and patients about SGLT-2 inhibitors, ensuring proper use, and avoiding unnecessary healthcare spending and drug misuse are crucial steps in maximizing their clinical and economic benefits. By comprehensively evaluating and optimizing implementation strategies, ensuring the global affordability and accessibility of SGLT-2 inhibitors can maximize their clinical and socio-economic benefits, benefiting more patients.

SGLT-2 inhibitors show great promise in treating non-diabetic CKD, but to fully realize their potential, continued efforts in multiple areas are required. In-depth mechanistic studies will help uncover their mechanisms of action in various pathological states and identify new therapeutic targets. Long-term studies will provide solid data to support their safety and efficacy in different populations (109). The development of new indications extends their range of application, bringing additional clinical benefits. Evaluating the economic and social impact and optimizing implementation strategies ensures their global affordability and accessibility. Through multidimensional research and collaboration, overcoming existing challenges, SGLT-2 inhibitors hold the potential to provide significant clinical benefits for more non-diabetic CKD patients, advancing the treatment of CKD and related chronic diseases, and improving patients’ quality of life (165).

## 6 Conclusion

SGLT-2 inhibitors, as multifunctional drugs, have demonstrated exceptional renal and cardiovascular protective effects in the treatment of non-diabetic CKD, significantly expanding their clinical application and value. Through multiple mechanisms, SGLT-2 inhibitors effectively reduce glomerular pressure, decrease proteinuria, and significantly delay the progression of kidney function deterioration through anti-inflammatory and antifibrotic effects. These effects have been thoroughly validated in key clinical trials such as DAPA-CKD, CREDENCE, and EMPA-KIDNEY, demonstrating their broad applicability and significant efficacy across different patient populations.

First, SGLT-2 inhibitors re-establish the tubulo-glomerular feedback mechanism, significantly lowering glomerular pressure and filtration rate, thereby alleviating the mechanical damage caused by hyperfiltration. This mechanism is effective in both diabetic and non-diabetic CKD patients, demonstrating its broad applicability across different pathological conditions. Additionally, by reducing proteinuria, SGLT-2 inhibitors not only alleviate the pressure on the glomerular filtration barrier but also inhibit related inflammatory and fibrotic responses, further protecting renal structure and function. Secondly, the anti-inflammatory and antifibrotic mechanisms of SGLT-2 inhibitors offer a fresh perspective for their use in CKD treatment. By regulating key signaling pathways such as AMPK, HIF-1α, TGF-*β*, and NF-κB, SGLT-2 inhibitors effectively reduce inflammatory responses and fibrosis in kidney tissues, thereby delaying further kidney function deterioration. The synergistic action of these multiple mechanisms not only enhances the drug’s renal protective effects but also provides potential applications for the treatment of other related diseases. In terms of overall metabolic improvement, SGLT-2 inhibitors reduce blood pressure, uric acid levels, and weight, improving patients’ metabolic state and significantly lowering cardiovascular disease risk. This comprehensive effect not only improves patients’ quality of life but also reduces the healthcare burden associated with metabolic syndrome-related complications. Particularly in the management of metabolic diseases such as obesity and hyperuricemia, SGLT-2 inhibitors have shown significant advantages, offering patients a more comprehensive and effective treatment option.

Although SGLT-2 inhibitors have demonstrated significant efficacy and broad potential in non-diabetic CKD patients, their use requires careful management of potential side effects, such as urinary tract infections, hypotension, and diabetic ketoacidosis. These risks can be effectively controlled and managed through appropriate patient selection, regular monitoring, and individualized treatment plans. High-risk populations, such as elderly patients, children, and those with a history of infections or metabolic disorders, require special attention and preventive measures to ensure the safe use of the drug.

Furthermore, the potential applications of SGLT-2 inhibitors in other kidney disease subtypes, such as IgA nephropathy, ADPKD, FSGS, and lupus nephritis, further broaden their clinical applications. By combining SGLT-2 inhibitors with RAAS inhibitors and antifibrotic drugs, significant synergistic effects are achieved, enhancing renal protection and delaying the progression of kidney disease. This multidrug combination therapy not only excels in controlling blood pressure and proteinuria but also shows unique advantages in inhibiting inflammation and fibrosis, offering a more comprehensive and effective treatment plan for CKD patients.

In future research and clinical practice, further exploration of the mechanisms of SGLT-2 inhibitors and long-term safety and efficacy evaluations in different populations and specific kidney diseases will be key to promoting their widespread use. Simultaneously, developing new indications and optimizing economic and social impacts will ensure that more patients benefit, improving the overall efficiency and effectiveness of the healthcare system. Through multifaceted research and international cooperation, SGLT-2 inhibitors are expected to be widely used globally, becoming an important drug choice in the management of CKD and related chronic conditions.

In conclusion, SGLT-2 inhibitors, in the treatment of non-diabetic CKD, not only significantly delay kidney function deterioration through multiple mechanisms but also improve overall metabolic and cardiovascular health, enhancing patients’ quality of life and prognosis. Their clinical significance cannot be underestimated, and as research deepens and applications expand, SGLT-2 inhibitors are poised to play an increasingly critical and widespread role in the treatment of CKD and related diseases, advancing modern nephrology and benefiting more patients.
